# Subacute cardiac rubidium-82 positron emission tomography (^82^Rb-PET) to assess myocardial area at risk, final infarct size, and myocardial salvage after STEMI

**DOI:** 10.1007/s12350-016-0694-x

**Published:** 2016-10-14

**Authors:** Adam Ali Ghotbi, Andreas Kjaer, Lars Nepper-Christensen, Kiril Aleksov Ahtarovski, Jacob Thomsen Lønborg, Niels Vejlstrup, Kasper Kyhl, Thomas Emil Christensen, Thomas Engstrøm, Henning Kelbæk, Lene Holmvang, Lia E. Bang, Rasmus Sejersten Ripa, Philip Hasbak

**Affiliations:** 1 Department of Clinical Physiology, Nuclear Medicine & PET and Cluster for Molecular Imaging, Rigshospitalet and University of Copenhagen, Copenhagen, Denmark; 2 Department of Cardiology, The Heart Center, Rigshospitalet Copenhagen University Hospital, Blegdamsvej 9, 2100 Copenhagen, Denmark

**Keywords:** Area at risk, final infarct size, myocardial salvage, rubidium-82 PET, SPECT, cardiac magnetic resonance

## Abstract

**Background:**

Determining infarct size and myocardial salvage in patients with ST-segment elevation myocardial infarction (STEMI) is important when assessing the efficacy of new reperfusion strategies. We investigated whether rest ^82^Rb-PET myocardial perfusion imaging can estimate area at risk, final infarct size, and myocardial salvage index when compared to cardiac SPECT and magnetic resonance (CMR).

**Methods:**

Twelve STEMI patients were injected with ^99m^Tc-Sestamibi intravenously immediate prior to reperfusion. SPECT, ^82^Rb-PET, and CMR imaging were performed post-reperfusion and at a 3-month follow-up. An automated algorithm determined area at risk, final infarct size, and hence myocardial salvage index.

**Results:**

SPECT, CMR, and PET were performed 2.2 ± 0.5, 34 ± 8.5, and 32 ± 24.4 h after reperfusion, respectively. Mean (± SD) area at risk were 35.2 ± 16.6%, 34.7 ± 11.3%, and 28.1 ± 16.1% of the left ventricle (LV) in SPECT, CMR, and PET, respectively, *P* = 0.04 for difference. Mean final infarct size estimates were 12.3 ± 15.4%, 13.7 ± 10.4%, and 11.9 ± 14.6% of the LV in SPECT, CMR, and PET imaging, respectively, *P* = .72. Myocardial salvage indices were 0.64 ± 0.33 (SPECT), 0.65 ± 0.20 (CMR), and 0.63 ± 0.28 (PET), (*P* = .78).

**Conclusions:**

^82^Rb-PET underestimates area at risk in patients with STEMI when compared to SPECT and CMR. However, our findings suggest that PET imaging seems feasible when assessing the clinical important parameters of final infarct size and myocardial salvage index, although with great variability, in a selected STEMI population with large infarcts. These findings should be confirmed in a larger population.

## Introduction

Reperfusion therapy has significantly reduced mortality in patients with acute myocardial infarction (AMI). Consequently, large numbers of patients are required to demonstrate further improvement in survival with introduction of new treatment strategies.^1^ Therefore, surrogate end-points for mortality are needed in proof-of-concept trials assessing the efficacy of new cardioprotective strategies. It has been shown that the most critical determinant of prognosis and outcome in patients with AMI is the final infarct size (FIS).^2^ The area at risk (AAR), which is the initial endangered myocardium, is a major determinant of the FIS,^3^ and therefore recommended to measure in order to risk stratify the patients.^4^ Measuring AAR and FIS enables determining the myocardial salvage index (MSI), which provides an indicator of therapeutic benefit.

Single-photon emission computed tomography (SPECT) has been extensively validated in clinical settings for the measurement of FIS and AAR, and is considered gold standard in determining AAR.^1^,^2^,^4^–^8^ The primary limitation of SPECT is that it is not easily applied in the clinical setting. Therefore, other methods have been developed such as cardiac magnetic resonance imaging (CMR), ECG-based scoring systems, and angiographic scores.^9^,^10^

CMR appears superior to SPECT in detection and quantification of infarct size, and CMR can also be used to assess AAR.^1^,^11^,^12^ However, CMR has additional contraindications compared to SPECT, most of all the presence of pacemakers and implantable defibrillators, claustrophobia, and renal insufficiency.

Another promising modality for measuring AAR and FIS is myocardial perfusion imaging with positron emission tomography (PET). Generator-based Rubidium-82 (^82^Rb) has now eased the access to myocardial perfusion with PET.^13^ Post-reperfusion imaging to depict AAR with PET might be feasible since ischemia/reperfusion injury entails a decrease in the sodium-potassium pump activity, hence limiting the incorporation of ^82^Rb in the myocytes in the endangered myocardium.^14^,^15^ With higher spatial and temporal resolution than SPECT and considerably shorter scan time than CMR and no contraindication, PET could provide accurate and reproducible measurements of AAR and FIS.

The aim of this prospective study was therefore to compare rest ^82^Rb-PET myocardial perfusion imaging to SPECT and CMR in terms of measurements of AAR, FIS, and MSI in patients with ST-segment elevation myocardial infarction (STEMI) undergoing reperfusion with primary percutaneous coronary intervention (pPCI).

## Methods

### Study group

**Figure 1:**
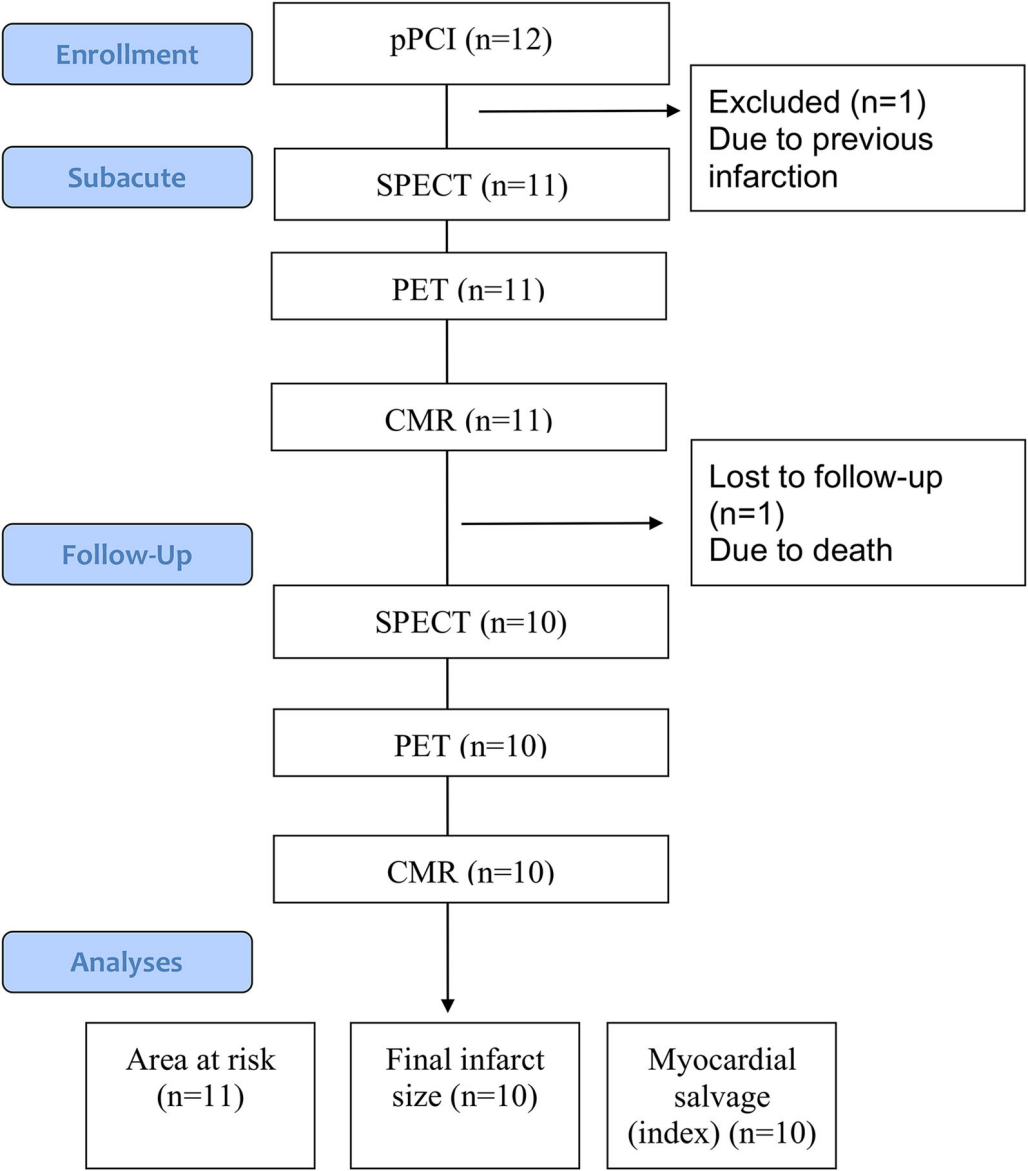
Study flow diagram. *pPCI* primary percutaneous coronary intervention; *SPECT* single photon mission computed tomography; *PET* positron emission tomography; *CMR* cardiac magnetic resonance

The study design is outlined in Figure 1. Twelve patients (11 male, median [interquartile range, IQR] age 58 (53; 68) years) with STEMI were enrolled. Duration from onset of symptoms to arrival at the catheterization laboratory was less than 12 hours. STEMI was defined as ST-segment elevation in 2 contiguous electrocardiographic (ECG) leads of >0.1 mV in *V*_4_ − *V*_6_ or leads II, III, and aVR, or > 0.2 mV in lead *V*_1_ − *V*_3_. Patient enrolment only took place during the opening hours of the Department of Nuclear Medicine. Exclusion criteria were cardiogenic shock, previous myocardial infarction, stent thrombosis, unconsciousness, or previous coronary artery bypass grafting.

Coronary angiography was performed to confirm occlusion in the infarct-related artery, and pPCI was performed according to local standard procedures.

All patients triaged for pPCI were pre-treated with standard therapy, including oxygen, sublingual nitroglycerin, aspirin (300 mg), prasugrel (60 mg), and heparin (10,000 units i.v.), and treated during the procedure with bivalirudin if not contraindicated.

### Ethics

The independent local ethics committee approved this study, protocol no: H-4-2010-054. All patients received oral and written information, and written consent was obtained from all patients before inclusion in compliance with the Declaration of Helsinki.

### SPECT acquisition

Prior to opening of the occluded vessel(s), patients received a 700 MBq i.v. injection of ^99m^Tc-Sestamibi. Myocardial SPECT imaging was performed within 1-4 hours after pPCI to visualize AAR. Patients were in supine position with continuous ECG monitoring. Images were acquired using a dual-head gamma camera with low-energy, high-resolution collimators, (Philips Precedence 16 Slice SPECT/CT, Eindhoven, The Netherlands) in an ECG-gated 64-by-64 matrix with 32 projections, acquisition time of 20 seconds per projection, and 8 frames per cardiac cycle with a 20% window centered on the 140 keV photopeak of ^99m^Tc. For attenuation correction, a low-dose computed tomography scan (CT) was acquired. Processing and reconstruction of SPECT images were conducted by iterative ordered subsets expectation maximization (OSEM) algorithm; 2 iterations, 10 subsets. The early SPECT imaging was considered as the gold standard of AAR estimation.^5^,^16^,^17^ A follow-up scan was conducted in a similar manner 3 months later to assess FIS. MSI was calculated as (AAR - FIS)/AAR.

### CMR acquisition

Patients were screened for contraindications, and if none existed, cardiac imaging was performed on a 1.5 T scanner (Avanto, Siemens, Erlangen, Germany) with the use of a 6-channel body array coil. CMR was conducted twice: subacute and at 3-month follow-up. The subacute imaging was not performed earlier than 12 hours after pPCI to allow for development of myocardial oedema, and < 3 days after pPCI. Patients were scanned in a supine position, and images were obtained at end-expiratory breath hold with ECG gating. To visualize edema and determine AAR (subacute scan only), multiple T2-weighted short tau inversion recovery sequences (slice thickness, 8 mm; field of view, 300-360 mm; inversion time, 180 ms; repetition time, 2 R-R intervals; echo time, 65 ms; slice gap, 0 mm) were applied in short-axis image plane from base to apex covering the entire left ventricle (LV). FIS was assessed at follow-up scan 10 minutes after the administration of diethylenetriamine pentaacetic acid (0.1 mL/kg; Gadovist, Bayer Schering, Berlin, Germany). An ECG-triggered enhancement inversion recovery was utilized (slice thickness, 8 mm; field of view, 300-360 mm; echo time, 1.4 ms, slice gap 0 mm). The LV was covered from base to apex in short-axis image plan: by adjusting the inversion time, the signal from the normal myocardium was nulled for each slice. The follow-up CMR FIS was, in this study, considered as gold standard method to estimate this parameter.^5^,^18^,^19^

### PET acquisition

Approximately, 24 h after ^99m^Tc injection, a rest cardiac PET imaging was performed using a Siemens Biograph mCT/PET 64-slice scanner (Siemens Medical, Knoxville, USA.) First, a low-dose CT scan was acquired for attenuation correction. Following i.v. administration of approximately 1,100 MBq of ^82^Rb (Cardiogen∙82^®^, Bracco Diagnostics Inc., Princeton, NJ, USA), dynamic and gated (8 frames per cardiac cycle) data acquisition was performed in 3D list mode for 7 min at rest. Images were reconstructed into 21 frames (12 × 10, 3 × 20, 6 × 30 seconds) with attenuation, scatter, and decay corrections using 3D OSEM, Gaussian filtering with 10 mm full width at half maximum. The follow-up scan was carried out 3 months later with similar settings to gauge FIS.

### SPECT and PET image analysis

Subacute and follow-up semi-quantitative data from SPECT and PET were both processed and analyzed semi-automatically in Cedars QPS/QGS^®^ software (v. 2012, Cedars Sinai, Los Angeles, CA, USA). Two experienced observers assessed the accuracy of slice alignments in the ventricular planes and intervened if necessary (blinded to CMR data). The perfusion defects (and thus AAR and FIS) were subsequently quantified in (1) the total LV, and (2) in each of the 17 segments according to the American Heart Association (AHA) 17-segment model.^20^ The magnitude of the rest perfusion defects (that was equal to assumed AAR in the subacute and FIS in the follow-up scan) was determined automatically by comparing the polar plot of a patient to that of the normal database on a pixel-by-pixel basis. A 2.5 standard deviation cut-off was used to define whether a pixel count fell below a normal value. The normal limit approach has previously been used to estimate AAR and FIS.^21^–^23^ However, we also estimated AAR in SPECT images using the threshold approach of 50% of peak counts and compared it to the results of the normal limit method.

The cut-off value of 2.5 standard deviations to define abnormality on the ^82^Rb PET uptake are derived from SPECT guidelines, but has not been validated for AAR assessment with ^82^Rb PET. Consequently, percentage of perfusion defect from PET in each of the 17 segments was compared to the gold standard of SPECT AAR in the segments to obtain optimal cut-off values with receiver operating characteristics (ROC) analysis.

### CMR image analysis

Endocardial and epicardial contours were manually traced in all short-axis images by two experienced observers (blinded to SPECT and PET data). A region of interest (ROI) was drawn in the normal (remote) myocardium, and AAR was defined as hyperintensive myocardium 2 standard deviations above the mean value in ROI on the T2-weighted images.^7^ Hypointensive areas within the AAR (e.g., hemorrhage or microvascular obstruction) were considered as part of the AAR. Scattered areas of hyperintensity in the normal myocardium were manually excluded. AAR was calculated as percent of the LV volume. Identical to the subacute scan, the endo- and epicardial contours were manually traced at the follow-up images, and a ROI was placed in the normal myocardium. FIS was defined as hyperintensive myocardium 5 standard deviations above the mean value in ROI.^7^ FIS was calculated as percent of the LV volume. The analyses were performed with CVI42 software, v. 4.0 (Circle Cardiovascular Imaging Inc., Calgary, Canada).

### Statistical analysis

Descriptive patient parameters are presented as median with IQR. Outcome variables are presented as mean ± standard deviation (SD) and categorical variables as frequencies or percent (%). All variables were tested with normality plots. To compare the three modalities, non-parametric Friedman test was used, and whenever a significant difference was observed, Dunn’s test for correction of multiple comparisons was performed. Correlation between any two modalities was examined by Spearman’s correlation. In addition, Bland-Altman test was performed to evaluate the agreement between SPECT, CMR, and PET. ROC analyses were generated in order to acquire the ideal cut-off values of PET parameters vs SPECT (“gold standard”). Accuracy, sensitivity, specificity, positive predictive value (PPV), and negative predictive value (NPV) were calculated for PET AHA 17-perfusion defects. A *P* value <.05 was considered significant. All statistical analyses were performed using SPSS^®^ version 19 (IBM, Chicago, IL, USA).

### Statistical considerations

In a previous study comparing CMR and SPECT, AAR and FIS have been presented as 30 ± 19% and 15 ± 17% of the LV with SPECT and 28 ± 15% and 16 ± 14% of LV with CMR (mean ± SD), respectively.^16^ We estimated the sample size in a pre-trial power calculation (*α* = type I error at 5% and 80% power (1 − β)) by two methods with the following:

1. *Sample size by correlation coefficient*: A correlation of no less than *r* = *0.75* would be acceptable; hence, a sample size of 11 paired patients was required.
2. *Sample size for paired difference in mean*: Prior investigations of SPECT and CMR claim a SD of 8-12 for mean difference. Thus, an acceptable mean difference of 8 ± 8% would result in a sample requirement of 10. To account for loss of patients to follow-up, 12 patients were included in the comparison of SPECT, PET, and CMR in regard to AAR and FIS.

## Results

Eleven of the initial twelve patients were included in the AAR analysis (one patient excluded due to previous infarction) and ten patients were included in the follow-up analysis (one patient died during follow-up) (Figure 1). Baseline characteristics are shown in Table 1.

**Table 1:** Baseline characteristics

	Patients (*n* = 11)
Age (years)	58 (53; 68)
Male	10 (91%)
Hypertension	2 (18%)
Hypercholesterolemia	2 (18%)
Total cholesterol, mmol/L	4.9 (4.1; 5.3)
Diabetes	0
Smoking	
Non	5 (46%)
Active	2 (18%)
Ex	4 (36%)
Family history of premature CAD	4 (36%)
Peripheral Arterial Disease	0
Infarct location	
LAD	6 (55%)
RCA	5 (45%)
LCX	0
TIMI flow prior to pPCI	
0	5 (46%)
1	3 (27%)
2	2 (18%)
3	1 (9%)
TIMI flow post-pPCI	
0	0
1	0
2	3 (27%)
3	8 (73%)
Peak Troponin T (ng/mL)	3710 (1450; 5850)
Peak CK-MB (U/I)	200 (70.9; 320)
Left Ventricle Ejection Fraction post-pPCI (echocardiography) (%)	40 (35; 50)
Time from symptom-onset to PCI (min)	175 (125; 300)
Time door-to-PCI (min)	27 (24; 29)

Values are median (interquartile range) *o*r n (%)

*CAD,* coronary artery disease; *LAD*, left anterior descending artery; *RCA*, right coronary artery; *LCX*, left circumflex artery; *TIMI*, thrombolysis in myocardial infarction; *CK-MB*, creatine kinase myocardial band; *pPCI*, primary percutaneous intervention

Median AAR estimation in SPECT images was not significantly different when measured with the threshold or the normal limit approach. Bias was −1.49 ± 12.8%, 95% limits of agreement were −26.5% to 23.5% (results not shown).

### SPECT, CMR, and PET comparison

**Figure 2:**
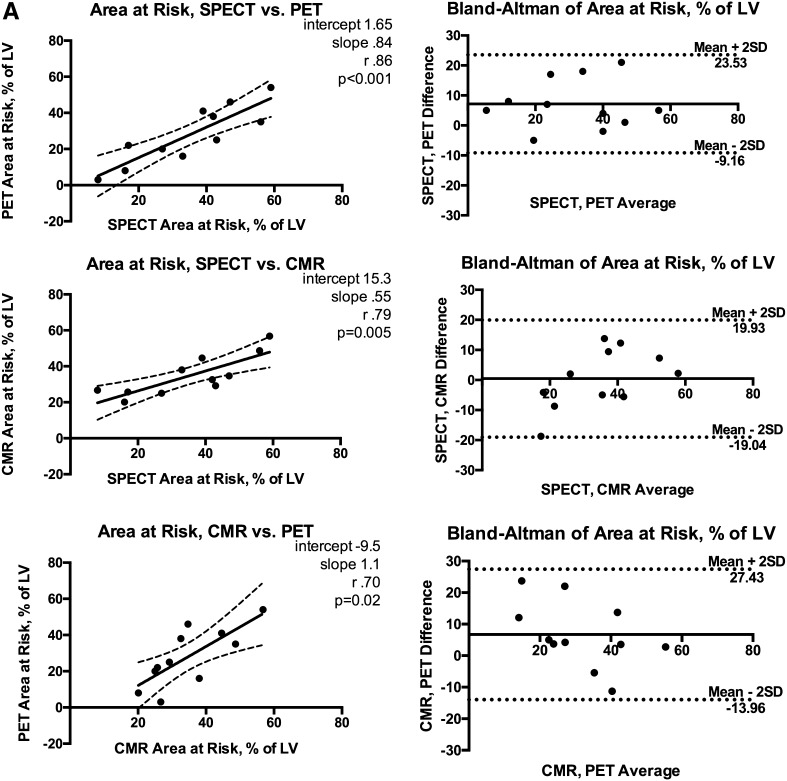

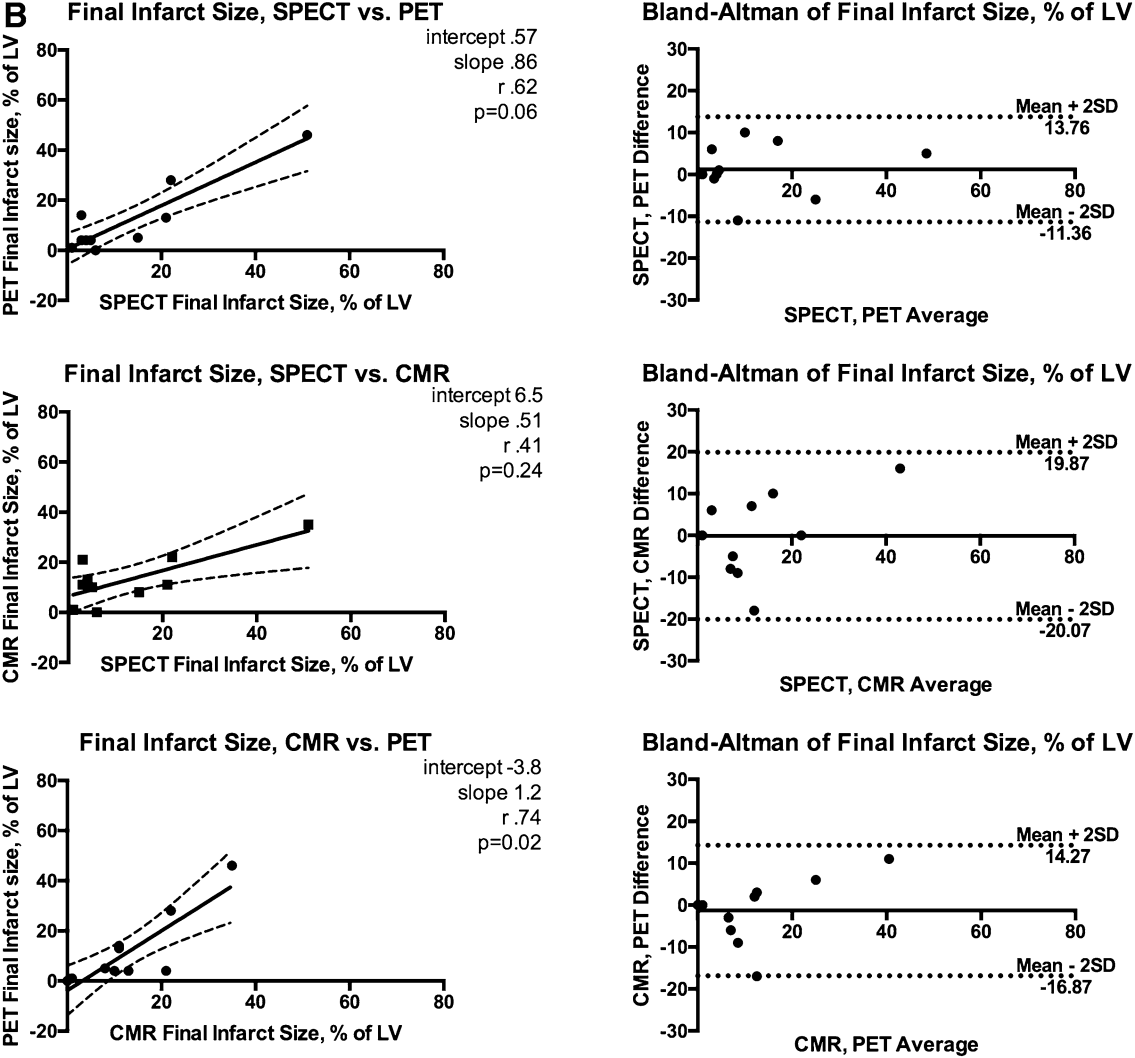

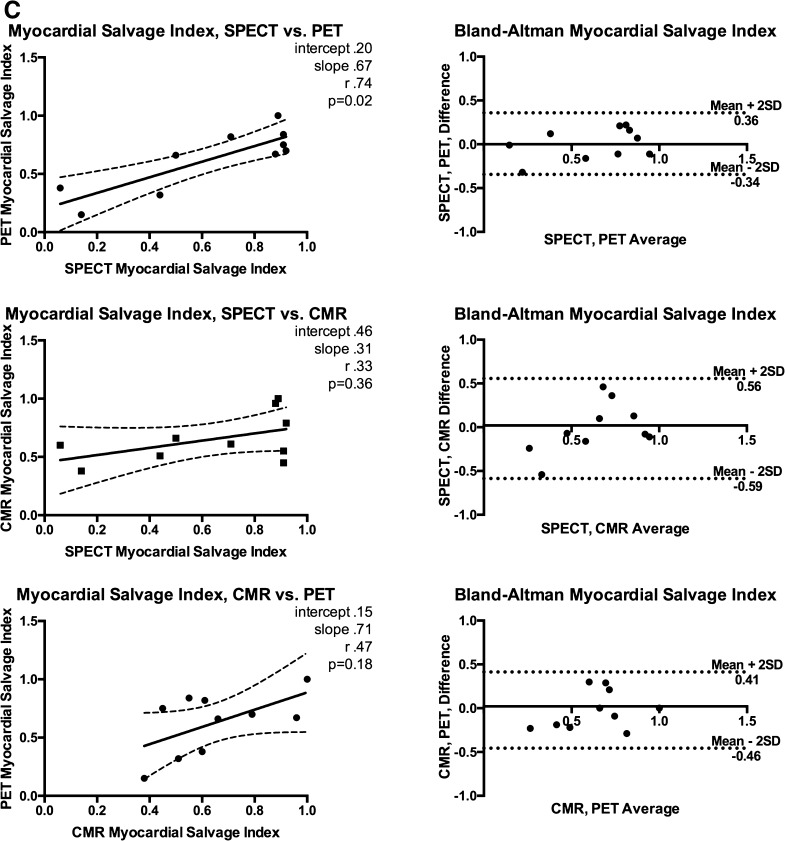
(**A**) Area at risk correlations, Bland-Altman plots. (**B**) Final infarct size correlations, Bland-Altman plots. (**C**) Myocardial salvage index correlations, Bland-Altman plots. *r*_s_ Spearman’s rho; *SD* standard deviation. Other abbreviations as in Fig. 1

SPECT, CMR, and PET were performed 2.2 ± 0.3 h, 34 ± 8 h, and 32 ± 7 h after ^99m^Tc-Sestamibi injection and pPCI, respectively. In SPECT imaging, mean AAR estimate was 35.2 ± 16.6%, and in CMR, AAR was 34.7 ± 11.3%, while in PET, AAR estimate was 28.1 ± 16.1% of the LV, resulting in a significant difference between the three modalities (*P* = .04). Post hoc paired tests revealed no significant difference between SPECT and CMR AAR (*P* = .75), whereas PET AAR estimate was significantly smaller compared to the other two modalities (*P* = .02 vs SPECT, *P* = .04 vs CMR). The 95% limits of agreement were −9.2 to 23.5% (SPECT vs PET), −19.0% to 19.3% (SPECT vs CMR), and −13.9% to 27.43% (CMR vs PET). SPECT correlated well in regard to AAR with PET and CMR (Spearman’s rho *r*_s_ = 0.86, 95% CI 0.51-0.96, *P* < .001; *r*_s_ = 0.79, 95% CI 0.35-0.95; *P* < .005, respectively) (Figure 2A).

Despite the overall good agreement between the 3 modalities, there were substantial differences in individual cases.

Follow-up SPECT, CMR, and PET were performed on the same day on average 95 ± 6 days after the initial pPCI treatment. Mean FIS estimate was 12.3 ± 15.4%, 13.7 ± 10.4%, and 11.9 ± 14.6% of LV in SPECT, CMR, and PET imaging, respectively, *P* = .72 for difference. 95% limits of agreement were −11.4% to 13.8% (SPECT vs PET), −20.1 to 19.9% (SPECT vs CMR), and −16.9% to 14.3% (CMR vs PET). Figure 2B depicts the agreement and correlation for follow-up measurements. MSI was comparable: 0.64 ± 0.33 (SPECT), 0.63 ± 0.28 (PET), and 0.65 ± 0.20 (CMR) with no statistically significant difference (*P* = .78) between the modalities; however, the correlations were weak and non-significant between CMR and the other modalities. Correlations, agreements, and MS indices are reported in Figure 2C and Table 2, respectively.

**Table 2:** SPECT, CMR and PET results

	SPECT	CMR	PET	*P* value (Friedman)
Time from ^99m^Tc tracer inj. to (h)	2.2 ± 0.3	34.5 ± 8.5^‡^	32.4 ± 24.4^‡^	0.02
Area at risk, % of LV	35.2 ± 16.6	34.7 ± 11.3	28.1 ± 16.1^‡§^	0.03
Final infarct size, % of LV	12.3 ± 15.4	13.7 ± 10.4	11.9 ± 14.6	0.72
Myocardial salvage index	0.64 ± 0.33	0.65 ± 0.20	0.63 ± 0.28	0.78

Values are mean ± SD

^*99m*^ *Tc*, technetium-99m; *LV*, left ventricle; *Salvage index*, (AAR-FIS)/AAR; *AAR*, area at risk; *FIS*, final infarct size

^‡^ *P* < .05 compared to SPECT

^§^ *P* < .05 compared to CMR

### Optimization of PET for accurate AAR assessment

A ROC curve was created to assess the discriminatory ability of PET-derived perfusion to detect SPECT AAR. The accuracy of PET AAR could be optimized by changing the segmental cut-off value of perfusion deficit to 35%, which resulted in a sensitivity of 85%, specificity of 94%, PPV of 87%, NPV of 93%, and accuracy of 91%. Area under the ROC curve was 0.92 (CI: 0.87-0.97, *P* < 0.0001).

## Discussion

To our knowledge, the present study is the first to compare and evaluate the use of PET to measure AAR, FIS, and MSI to the current gold standard methods of SPECT and CMR. Despite differences in tracer property, imaging technique, reconstruction algorithms, and intervening revascularization, the three modalities correlated well in regard to AAR and FIS. However, the limits of agreement were fairly large, and PET underestimated the AAR with approximately 7% compared to SPECT. Our data suggest that this difference could be corrected for by applying new PET cut-off values to distinguish normal from hypoperfused segments. However, this cut-off is exploratory and needs to be verified in a separate cohort. Overall, the clinical relevant parameters of FIS and MSI were comparable between the modalities.

It is of great importance to establish the AAR when evaluating the efficacy of new infarct-limiting strategies because the variation in the endangered area is great even with similar segments with coronary occlusion.^24^ However, the concept and definition of AAR has recently gained attention since no clear standardized measurement exists.^25^,^26^ The original SPECT-derived AAR measurements are based on pioneer studies from late 1980s and early 1990s.^8^,^27^ These studies used reconstruction techniques that are different form current practice (i.e., filtered back projection without AC). Recently, the T2-weighted method of delineating AAR by CMR has come under criticism. Kim et al. argue that the developed edema does not depict AAR but rather the infarct size.^28^ Therefore, the concept of “gold standard” must be viewed with caution.^29^

Not surprisingly, the estimated AAR was significantly smaller in PET imaging compared to SPECT and CMR. SPECT and PET imaging assess perfusion with different approaches. While ^99m^Tc-Sestamibi is incorporated in the mitochondria of living myocytes, ^82^Rb works as a potassium analogue and accumulates in the myocytes via the Na+/K+ ATPase.^13^ Furthermore, fundamental differences in image acquisition and technology between SPECT and PET could, at least in part, explain the differences in measured AAR. Previously, different cut-off values of 50%^30^ and 60%^8^ of peak counts have been proposed as the optimal cut-off to depict AAR with SPECT. We choose a similar cut-off value for SPECT and PET, 2.5 SD (≈50%). However, we find that an optimization of the cut-off values between normal and hypoperfused myocardium is possible with PET. Although earlier papers have used the normal limits approach to measure the AAR,^21^–^23^ the method has not been vastly validated and could pose a limitation.

Previous papers^16^,^17^ have reported smaller AAR estimations by SPECT and CMR than our results. This could potentially result in an overestimation of MSI. The discrepancies could be the result of our small sample population and selection bias. However, other studies report comparable CMR-derived AAR estimations and standard deviations to our results.^31^,^32^

It is somewhat counterintuitive that ^82^Rb-PET after revascularization can assess the AAR. The potential mechanism is unknown, but it is our hypothesis that although the patency of an epicardial artery is re-established, the ischemia/reperfusion injury may entail microvascular impairment^33^ and depress the myocytes Na+/K+ ATPase activity.^15^,^34^ This damage to the coronary microcirculation and the decrease in activity in the sodium-potassium pump could explain the defects seen subsequent to pPCI and enables ^82^Rb to visualize AAR. In addition, the contrast between previously jeopardized myocardium (AAR) and normal myocardium after AMI seems enhanced by findings of hyperaemia in the normal myocardium only.^35^,^36^ It may be speculated that the decreased flow in the infarct-related territory, compared to normal myocardium, is a manifestation of microvascular obstruction/dysfunction due to edema, clotting by blood components, and endothelial disruption.^37^

Serial ^99m^Tc SPECT imaging before and 18-48 h after reperfusion therapy has previously been conducted to demonstrate patient infarct-related artery when treated with thrombolytic agents.^38^–^40^ They reported marked reductions in the extent of defect size (9%-50%) between initial and follow-up imaging at 18-48 hours. It has been suggested that the uptake of ^99m^Tc after reperfusion not merely depends on blood flow but also the viability of the myocardium, thus reflecting the degree of necrosis and salvage.^41^ It seems that SPECT imaging at 18-48 hours measures AAR with a large difference compared to pre-reperfusion assessment.

FIS is considered an important surrogate marker of mortality and morbidity,^6^ and in many studies used as primary end-point.^22^,^42^,^43^ Median FIS was not significantly different when compared across the three modalities. PET had minor bias and a very close correlation with CMR. Despite the good agreement between PET and CMR, substantial differences and variability exist regarding the measurement of FIS, which is demonstrated by the large limits of agreement. Hadamitzky et al. showed similar large limits of agreement when comparing SPECT to CMR.^16^ It would be important if PET could estimate FIS comparable to CMR, since a considerable number of patients are unable to undergo CMR due to claustrophobia or other contraindications.^44^

MSI is of clinical importance, since it conveys a measurement of the potential benefit patients with AMI experience from a certain reperfusion therapy.^45^,^46^ MSI derived from the three modalities showed good congruence. It is noteworthy that in some studies,^43^,^45^ only MSI is a predictor of mortality and not myocardial salvage alone. Recently, a study demonstrated that MSI by CMR could reduce sample size in cardioprotection trials by 46% compared to myocardial infarction alone.^47^ However, the variability of MSI in our study was notable, although comparable to the results of Hadamitzky *et al*.^16^ Moreover, the correlations between the modalities were not significant when comparing CMR with PET or SPECT.

The financial aspects of the three modalities are beyond the scope of this paper, but the expenses associated with each scanner and the monthly cost of an ^82^Rb generator should be taken into consideration.

## Limitations

Due to the comprehensive study protocol, the number of patients included was small and may impact our conclusions due to risk of type II error. Furthermore, we lack stress-induced PET imaging, which could have provided additional information regarding coronary flow reserve. We did not perform stress imaging due to the proximity to the index STEMI of concern for adverse effects. Previous perfusion studies using N-13 ammonia early after MI did not reveal any significant difference in infarct size under resting and adenosine conditions, hence questioning the absolute need for stress imaging.^48^

AAR and FIS estimations in SPECT and PET imaging are dependent on the software applications in use, and there is no consensus on which application to use.^29^ The normal limit approach to estimate AAR and FIS in SPECT has not been used on regular basis and therefore not extensively validated, albeit some papers have previously applied this method.^21^–^23^ Furthermore, no standard technique is widely accepted for CMR quantification of AAR and FIS on late gadolinium and T2-weighted images, respectively.^49^ Thus, lack of a well-defined, explicit “gold standard” reference for both AAR and FIS could be argued to be a limitation.

## Conclusion

The present study suggests that determining FIS and MSI is feasible with ^82^Rb-PET imaging shortly after pPCI and at follow-up in a STEMI population with larger infarcts, albeit a vast variability hampers direct transference of results between the modalities. In addition, PET underestimated AAR with 7% compared to SPECT, but our data suggest that AAR assessment by PET could be optimized with the use of new cut-off values to define abnormality. These findings should be confirmed and further optimized in a larger patient STEMI population.

## New Knowledge Gained

^82^Rb-PET could potentially allow fast and reliable estimation of FIS and MSI, which are important parameters in evaluating new reperfusion strategies. With lower radiation than SPECT and no contraindication compared to CMR, ^82^Rb-PET could be an alternative in the post-infarction cardiac imaging toolbox.

## Abbreviations

AAR: Area at risk
AHA-17: American Heart Association 17-segment model
AMI: Acute myocardial infarction
CMR: Cardiac magnetic resonance
FIS: Final infarct size
LV: Left ventricle
MSI: Myocardial salvage index
OSEM: Ordered subsets expectation maximization
pPCI: Primary percutaneous coronary intervention
PET: Positron emission tomography
SPECT: Single-photon emission computed tomography
STEMI: ST-segment elevation myocardial infarction
^82^Rb: Rubidium-82
ROI: Region of interest
